# Deletion of 17p in cancers: Guilt by (p53) association

**DOI:** 10.1038/s41388-025-03300-8

**Published:** 2025-02-18

**Authors:** Francisca van Kampen, Abigail Clark, Jamie Soul, Aditi Kanhere, Mark A. Glenn, Andrew R. Pettitt, Nagesh Kalakonda, Joseph R. Slupsky

**Affiliations:** 1 Department of Molecular and Clinical Cancer Medicine, Institute of Systems, Molecular and Integrative Biology, University of Liverpool, Liverpool, UK; 2 Computational Biology Facility, University of Liverpool, Liverpool, UK

**Keywords:** Cancer genetics, Cancer genomics, Haematological cancer

## Abstract

Monoallelic deletion of the short arm of chromosome 17 (del17p) is a recurrent abnormality in cancers with poor outcomes. Best studied in relation to haematological malignancies, associated functional outcomes are attributed mainly to loss and/or dysfunction of *TP53*, which is located at 17p13.1, but the wider impact of deletion of other genes located on 17p is poorly understood. 17p is one of the most gene-dense regions of the genome and includes tumour suppressor genes additional to *TP53*, genes essential for cell survival and proliferation, as well as small and long non-coding RNAs. In this review we utilise a data-driven approach to demarcate the extent of 17p deletion in multiple cancers and identify a common loss-of-function gene signature. We discuss how the resultant loss of heterozygosity (LOH) and haploinsufficiency may influence cell behaviour but also identify vulnerabilities that can potentially be exploited therapeutically. Finally, we highlight how emerging animal and isogenic cell line models of del17p can provide critical biological insights for cancer cell behaviour.

## Introduction

Deletion of the whole or part(s) of the short arm of chromosome 17 (del17p) is a recurrent chromosomal aberration observed in many cancers [1–4]. del17p is particularly common in solid cancers such as pancreatic ductal adenocarcinomas and colorectal carcinomas where more than 60% of tumours have been reported to contain malignant cells bearing this copy number alteration (CNA) [1, 5–7]. Other cancers such as non-small cell lung cancer [8], prostate cancer [9], breast cancer [4] and squamous cell carcinoma of the cervix [10] also harbour this abnormality. Importantly, del17p is also observed in haemic cancers such as chronic lymphocytic leukaemia (CLL) [11], multiple myeloma (MM) [12], diffuse large B cell lymphoma (DLBCL) [13], mantle cell lymphoma (MCL) [14] and acute myeloid leukaemia (AML) [2], where it is associated with rapid progression, short survival and resistance to therapies [15, 16]. From a mechanistic point of view our understanding of the impacts of the loss of multiple genes is limited. The vast majority of studies investigating del17p in cancer have focussed on dysfunction of *TP53* which is located at 17p13.1. Whilst the p53 protein encoded by *TP53* has an important function in protecting cells from genomic and environmental insults, and in many cases is accompanied by nonsense and missense *TP53* mutations that lead to functional loss of this protein [17], studies of CLL [16, 18], and other cancers [1, 8, 9, 12, 19], have shown that del17p is associated with adverse prognosis even in the absence of p53 dysfunction. It is only recently that we have begun to appreciate the wider impact of del17p on cancer cells. The purpose of this review is to assess the impact of del17p on cell behaviour and suggest new horizons for future research and therapies.

Why is a better understanding of such impact important? Firstly, it is clear that the emergence of del17p is more common after DNA-damaging chemotherapy and/or radiotherapy and likely signifies clonal selection and outgrowth [20]. Such clones are invariably and inherently more resistant to further chemotherapy/radiotherapy. Secondly, even in diseases such as CLL, where targeted agents such as ibrutinib have largely replaced chemotherapeutic strategies, outcomes are significantly worse among patients with del17p, mutated *TP53* [21, 22] or complex karyotypes [23] associated with del17p [24]. Finally, a better understanding of cancer relevant haploinsufficiencies resulting from loss of heterozygosity (LOH) at 17p may identify as yet uncharacterised targets to inform novel therapies that exploit synthetic lethality.

## Anatomy of del17p

Chromosome 17 is highly susceptible to non-allelic homologous recombination due to its unusually complex rearrangement and duplication structure [25]. The presence of low copy number repeats, particularly in the short arm, foster this form of recombination to generate microdeletions of varying sizes. The best characterised are Miller-Dieker syndrome (MDS) and isolated lissencephaly sequence (ILS) which result from variable deletion of a critical 1.3 Mbp region within band 17p13.3 spanning between the essential genes *PAFAH1B1* and *YWHAE* [26], and Smith–Magenis syndrome which in approximately 80% of cases is associated with microdeletion of a 3.7 Mbp region in band 17p11.2 [27]. The genes affected by these deletions lead to defects in craniofacial and brain development, as well as neurological sequelae such as epilepsy. Other microdeletion syndromes include hereditary neuropathy with pressure palsies which results from deletion of a chromosomal section in 17p12 containing *PMP22* [28], as well as 17p13.1 and 17p13.2 microdeletion syndromes [29, 30]. These genetic conditions usually arise spontaneously in an autosomal dominant fashion, and range in severity with respect to gene expression and clinical impact. Importantly, microdeletions within 17p13.3 and 17p13.2 tend to be more severe such that affected individuals rarely reach adulthood [26, 29]. In contrast, the impact of microdeletions within 17p13.1, 17p12 and 17p11.2 in affected individuals are milder and do not curtail life expectancy [27, 30]. Importantly, these autosomal microdeletions of 17p do not appear to increase the risk of cancers in affected individuals [30].

**Fig. 1:**
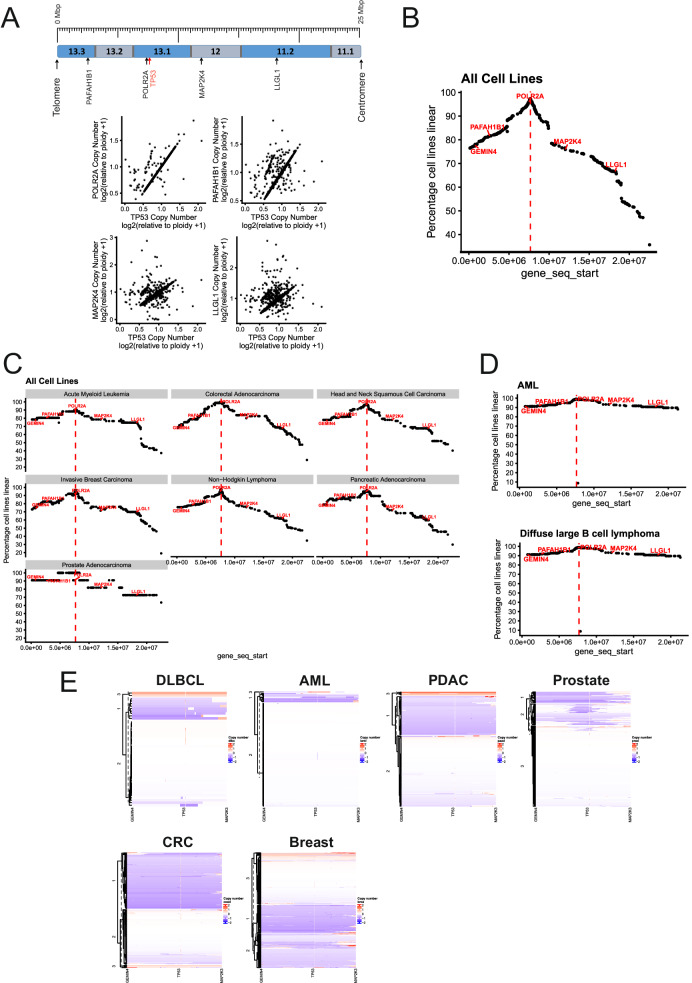
Analysis of deletion size associated with 17p aneuploidy in various cancers. **A** Dot plots showing copy number alteration (CNA) data of the four indicated genes (upper panel shows where they are located on chromosome 17p) plotted relative to *TP53* CNA. The CNA data is derived from the cancer cell lines available on DepMap. **B** Graph showing the frequency of genes located on 17p that are co-deleted with *TP53* in a continuous fashion, giving insight into the size of deletion that is most commonly observed in cells bearing del17p. The location of *TP53* is indicated by the dotted red line, as are the positions of the genes illustrated in part A. The data used to construct this graph is taken from all cell lines available on DepMap. **C** Graphs showing the frequency of genes located on 17p that are co-deleted with *TP53* in a continuous fashion using data (from DepMap) associated with cell lines derived from the indicated cancers. The location of *TP53* is indicated by the dotted red line, as are the positions of the genes illustrated in part A. **D** Graphs showing the frequency of genes located on 17p that are co-deleted with *TP53* in a continuous fashion using data (from cBioPortal) associated with primary cells derived from the indicated cancers. The location of *TP53* is indicated by the dotted red line, as are the positions of the genes illustrated in part A. **E** Hierarchical cluster plots indicating 17p deletion size (x-axis) within cells from individual patients (y-axis) calculated from data recorded in cBioPortal for primary cells from the indicated cancers.

In contrast to microdeletions, macro-deletions of 17p within malignant cells span a wider genomic region that varies widely between individual cancers. To understand the nature of this variability we used data available on the DepMap (https://depmap.org/portal/) [31, 32] and cBioPortal (https://www.cbioportal.org/) [17, 33] platforms to investigate the linkage of CNA among genes located on 17p. Figure 1A shows the relationship between copy numbers of *TP53* (often used clinically as an indicator of 17p deletion) and of four other genes situated telomeric (*POLR2A*, *PAFAH1B1*) or centromeric (*MAP2K4*, *LLGL1*) to *TP53*. The high degree of concomitant loss, particularly of genes in the direct vicinity of *TP53*, strongly suggests co-deletion with *TP53* and that such CNA occurs in a continuous fashion. This notion is illustrated in Fig. 1B where further analysis of this relationship across all cell lines available on the DepMap portal shows that CNAs of genes located telomeric to *TP53* are more likely to show such concomitant loss than those that are centromeric. Furthermore, Fig. 1B also shows that the size of deletion is most likely to extend from GEMIN4 at the telomere to MAP2K4 close to the centromere. Application of this algorithm to cell lines derived from specific cancer types showed largely similar sizes of 17p loss with the exception of prostate adenocarcinoma where there was evidence for the occurrence of discreet microdeletions as opposed to the large-scale deletion observed associated with the other cancer cell lines (Fig. 1C). Large-scale deletion of 17p was also evident in AML and DLBCL using data obtained from primary patient samples within the cBioPortal platform (Fig. 1D), an observation made more evident by clustering the samples from individual patients. This showed that del17p is likely to encompass nearly the entire short arm in DLBCL, AML, breast, pancreatic and colorectal cancers (Fig. 1E). Importantly, this analysis also revealed the respective prevalence of del17p in patient samples from each of these tumours, revealing that this aneuploidy is more likely to be observed in solid than in haemic malignancies. With respect to prostate cancers, our findings were in keeping with those using cancer cell lines and del17p was manifested in about half of cases as microdeletions within 17p13.1 (Fig. 1E). Our findings are supported by studies reporting large deletions encompassing most of the entire arm of 17p in CLL [34], DLBCL [35], MM [36], AML [2] and some solid tumours [4–6, 9], typically using either SNP arrays or fluorescence in-situ hybridisation techniques such as chromosome painting. Key to the insight provided by the above analysis is that despite the much higher prevalence of del17p within solid tumours, our understanding of del17p and its impact on patient outcomes is mainly derived from studies of haemic malignancies.

## Is del17p the cause or result of genetic instability and/or p53 inactivation?

**Fig. 2:**
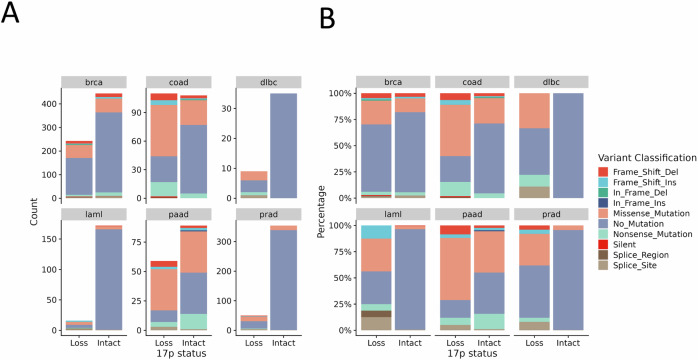
Analysis of 17p deletion in relation to mutation of *TP53*. Data from cBioPortal on mutation within *TP53* was analysed against the presence or absence of del17p for the indicated cancers (**brca** breast invasive carcinoma, **coad** colorectal adenocarcinoma, **dlbc** diffuse large B cell lymphoma, **laml** acute myeloid leukaemia, **paad** pancreatic adenocarcinoma, **prad** prostate adenocarcinoma) and illustrated according to **A** count within the total data set available, or as **B** a percentage within each variable (loss vs. intact 17p). *TP53* mutation classification is indicated by the colour scheme.

A large question that looms over the study of del17p is whether it is the cause or consequence of genetic instability and/or p53 inactivation. This is because del17p is often associated with tumours with high chromosomal instability (CIN) and/or with p53 which has become dysfunctional, and because del17p is not always associated with aggressive tumour behaviour; a study of CLL patient outcomes where del17p is observed at diagnosis reported the presence of cases where disease progression was slow [37]. However, slow disease progression in this situation could be the result of association with good prognostic indicators such as mutated *IGHV* genes and/or to the presence of a low percentage of cells with del17p and/or whether *TP53* was mutated as was reported by Yuan et al. [16] in a subsequent study of a larger cohort. This same study reported that presence of mutated *TP53* together with del17p correlated with the poorest clinical outcomes, and this brings the question of whether dysfunction of p53 is a driver of 17p loss. Zenz et al. [38] published a detailed mutational profile of *TP53* in CLL patient samples with and without del17p, noting that such mutation was present in 76% of cases. Such prevalence is consistent with other tumours such as AML and colorectal and pancreatic cancer (Fig. 2), but other malignancies, such as breast cancer and multiple myeloma, show considerably less, suggesting that association between mutated *TP53* and del17p may be cancer dependent. In this respect, *TP53* LOH may contribute to p53 inactivation due to haploinsufficiency. This effect has been shown in isogenic cell lines where monoallelic disruption of *TP53* expression leads to lower p53 mRNA and protein levels to impact p53-mediated gene expression [39]. Furthermore, one study investigating the relevance of del17p in cell lines derived from MM showed that *TP53* haploinsufficiency is associated with reduced p53 activation in response to etoposide or nutlin [40]. Regarding the effect of del17p in primary cancer cells, gene expression profiling revealed under-expression of p53 in CLL cells with a 17p deletion [41], but here it is important to note that p53 mRNA expression may be reduced by mechanisms other than *TP53* LOH [17, 33]. Nevertheless, a recent study demonstrated that the introduction of gain-of-function missense mutations into p53, notably R175H and R273H, resulted in increased levels of aneuploidy in recipient cell lines compared to those which received nonsense mutations [42]. Importantly, del17p was not the most common deletion observed within this experimental system. However, studies of fibroblasts derived from patients with Li-Fraumeni syndrome (LFS), an inherited disorder where germ-line mutations in *TP53* are of a similar nature to those reported by Redman-Rivera et al. do show LOH associated with the chromosome arm containing the wildtype allele of *TP53* as part of observed increased genomic instability [43, 44]. Thus, dysfunctional p53 can drive 17p deletion, but it seems missense mutations are favoured, a notion supported by the cancers we studied for this review where *TP53* with missense mutations are significantly represented in tumours with del17p (Fig. 2).

Is del17p a cause or consequence of genetic instability? Certainly dysfunction of p53 is a driver of such instability and there is reported association with del17p, complex karyotypes, and chromothripsis in CLL [24] and other cancers [45]. A recent study comparing patterns of chromosomal instability in colorectal cancers showed that 17p deletion occurs earlier in cases with high levels of chromosomal complexity compared to cases with lower levels [46]. These findings are in keeping with an earlier report investigating *TP53* inactivation and complex karyotype in CLL cells [47]. To this end, several genes on 17p are involved in maintaining genomic integrity. These include *RPA1*, *WRAP53* and *CTC1* which also play a direct role in maintaining telomere length, implying that del17p may also influence replicative senescence. In keeping with this, senescence was induced in cells by depletion of MIS12, a key protein encoded at 17p13.2 that is also involved in kinetochore formation [48]. *TOP3A*, encoded at 17p11.2 and part of the BTR (Bloom syndrome protein (BLM)–TOP3A–RecQ-mediated genome instability proteins (RMI1/2)) dissolvasome complex, is another gene affected by del17p that has a dual role in maintaining genomic integrity and preserving telomere length [49]. In CLL cells there is a reported increase in telomere sister chromatid exchange rates that is correlated with strong down-regulation of TOP3A expression [50]. Telomere dysfunction is a feature of progressive CLL [51] and this can lead to the generation of inter-chromosomal fusions, including with 17p, as reported by Escudero et al. [52]. Here, it is important to note that 17p may be particularly sensitive to the effects of telomere shortening because its telomere is the shortest within the genome [53]. This notion is supported by one report linking further shortening of the 17p telomere to chromosome instability and heteroallelic loss of 17p in Barrett’s oesophagus and development of cancer [54], and by others where short telomeres in general are linked to del17p and increased genetic complexity in CLL and Barrett’s oesophagus [54–58]. Thus, it would seem that telomere shortening is a driver of 17p deletion because of its known role as a driver of genetic instability in cells (reviewed by Maciejowski and de Lange [59]). However, within this context there is also requirement for loss of p53 function in order for cells to tolerate such instability [59], explaining why *TP53* mutation is so strongly associated with del17p and high CIN in cancers. Nevertheless, del17p can occur in cancers where p53 is wildtype (Fig. 2), and does feature as a singular aneuploidy in CLL and other tumour types. For this latter reason, this review will focus on the impact of del17p in cells.

## Potential cellular and biological consequences of 17p deletion – protein coding genes

**Fig. 3:**
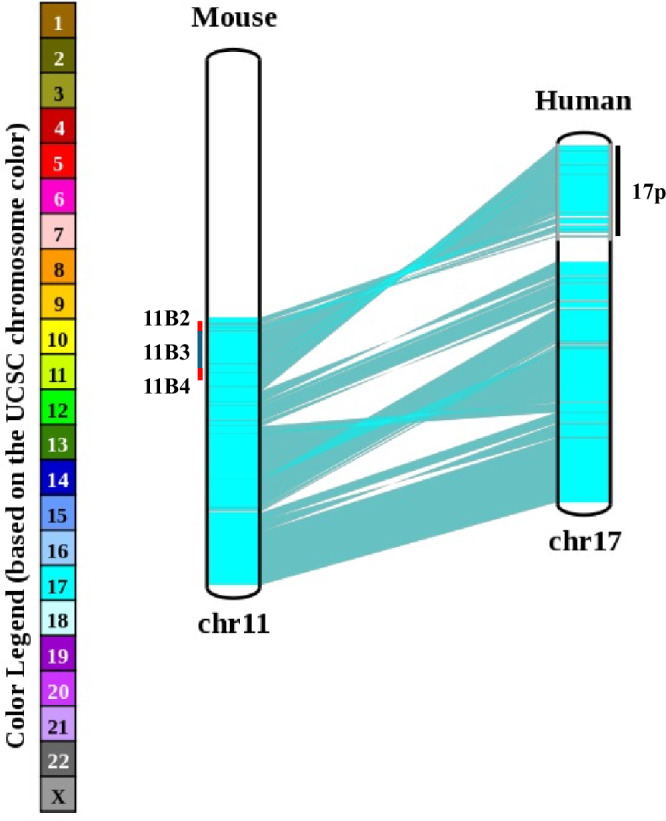
Human chromosome 17 is syntenic with mouse chromosome 11B. A chromosome map comparing gene arrangement between mouse chromosome 11B and human chromosome 17. The cytobands 11B2, 11B3 and 11B4 are marked for reference to human 17p.

Data obtained from OMIM and UCSC Genome Browser indicate that 17p contains 328 protein coding genes as well as 25 miRNAs, 292 lncRNAs, and 153 pseudogenes. Chromosome 17 is second only to chromosome 19 in terms of gene density [25], with bands 17p13.3 and 17p13.2 being rich in gene numbers. Importantly, human chromosome 17 shares high synteny with the distal approximate 60 Mb region of mouse chromosome 11, with cytobands 11B2 – 11B4 on mouse chromosome 11 being representative of human chromosome 17p (Fig. 3). This means that mouse models of del17p can be created if reported embryonic lethality can be overcome by induction of tissue-specific deletion as has been demonstrated in key papers [2, 60].

**Fig. 4:**
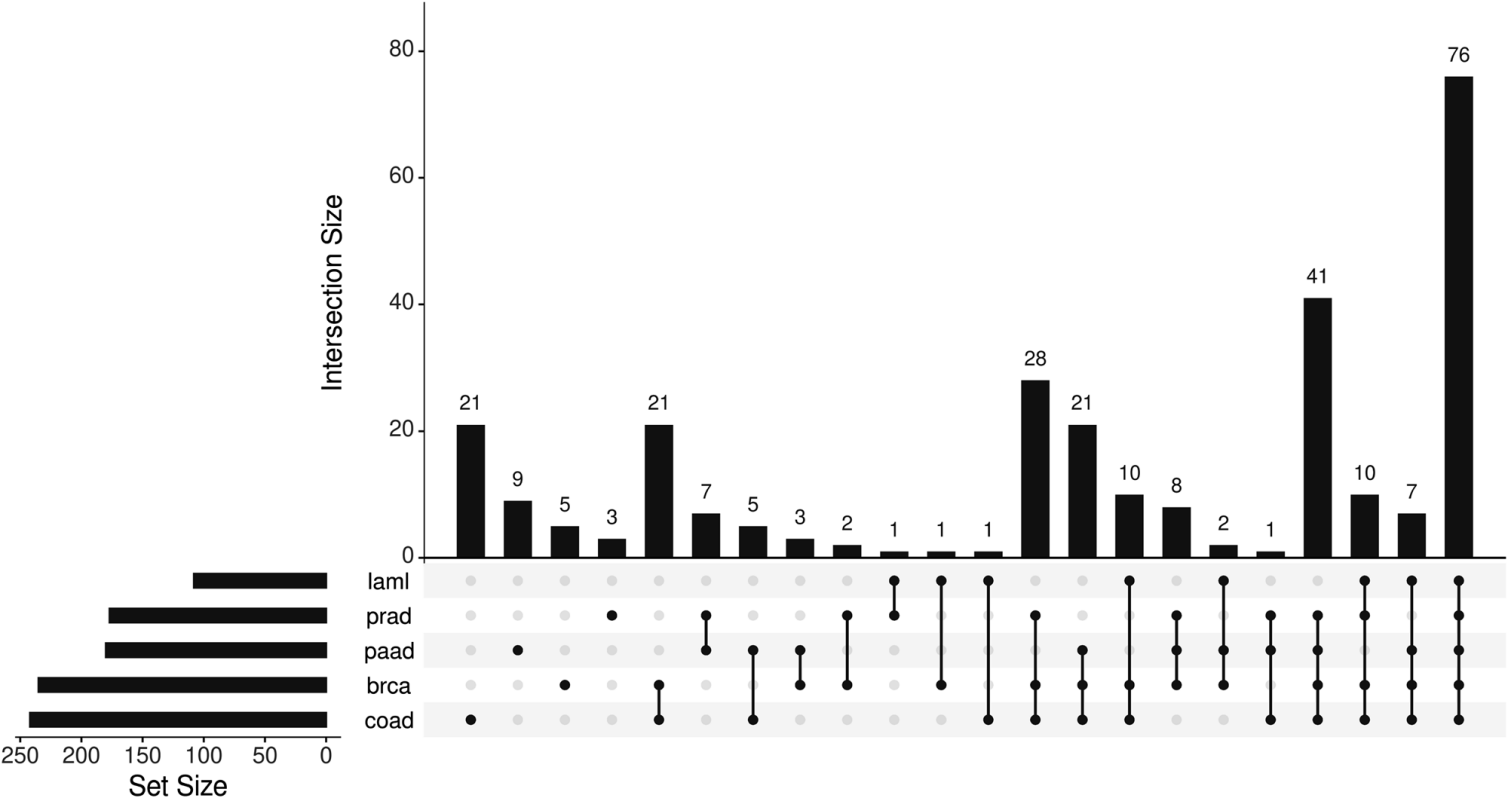
Upset analysis of gene expression in primary malignant cells with del17p. Gene expression in primary malignant cells with or without del17p was compared (with DESeq2), focusing on genes located on 17p that were downregulated in del17p cells. Set size refers to the number of genes showing change in cells from the indicated cancers. Intersection size refers to the shared genes between the different cancers analysed. Cells from 5 cancers were compared; **laml** acute myeloid leukaemia, **prad** prostate adenocarcinoma, **paad** pancreatic adenocarcinoma, **brca** breast invasive carcinoma, **coad** colorectal adenocarcinoma. The data for analysis was taken from cBioPortal.

An important question here is whether haploinsufficiency of genes other than *TP53* that are located on 17p is relevant for cancer biology. We previously investigated the gene expression profile of primary CLL cells obtained from patients with or without del17p and identified several genes in addition to *TP53* that were under-expressed in cases with del17p including *YWHAE*, *PRPF8*, *PAFAH1B1*, and *NUP88* [41]. Using a bioinformatics approach similar to the one reported by Li et al. [4] we examined TGCA data [17, 33] for genes located on 17p that are differentially expressed between samples with or without del17p obtained from patients with different types of cancer. An UpSet plot of this analysis revealed a panel of 76 genes, including the four we identified in CLL, that were consistently under-expressed, suggesting haploinsufficiency, in del17p samples from AML, colorectal, pancreatic, prostate and breast cancers (Fig. 4, Table 1). A significant feature of this panel is the inclusion of eighteen genes designated “common essential” as determined by CRISPR/Cas9 and siRNA screens of cancer cell lines [31, 32]. This designation relates to an absolute requirement of the protein produced by these genes for the survival of affected cells and, together with our observation of reduced expression of these genes in cells with del17p, suggests potential vulnerability. Indeed, haploinsufficiency with respect to expression of these genes has the potential of making cells bearing del17p less fit. This concept is largely unexplored with the exception of a study reporting the development of a tool, known as BISCUT, which was used to show correlation between cellular fitness and aneuploidy [61]. Although not relevant to del17p, this study identified *WRN*, a gene located on chromosome 8, as a haploinsufficient TSG able to drive tumour growth. This is important in the context of this review because 27 of 1217 recognised tumour suppressor genes (including *TP53*) are located on 17p at a significantly higher density than on other chromosome arms [62], and our analysis shows that 11 TSGs have lower expression in cells with del17p (Table 1). An additional notion to consider is that cells, including malignant cells, take cues from their environment that regulate their growth, a phenomenon known as cell competition (reviewed in Morata [63]). Here, cells with del17p may be outcompeted by other cancer cells that may be more fit in tumours that are heterogeneous, but this homoeostasis can rapidly change following interventions, such as when therapy is applied, leading to expansion and dominance of cell clones bearing del17p. The concept of cell competition could, therefore, be used to explain why del17p is not always associated with aggressive disease in CLL [37]. The proportion of malignant cells carrying del17p clone also plays a role where reports indicate that when this proportion is high, disease outcome in CLL [16] and MM [64] is poor and vice versa. The notion of increased vulnerability is supported by reports showing that reduced expression of *POLR2A* in del17p prostate and breast cancer cells is associated with enhanced sensitivity to the fungal toxin α-amanitin [4, 9]. Similarly, LOH and haploinsufficiency have been observed for the splicing factor *PRPF8* in AML where reports indicate that reduced expression of this gene lead to increased proliferation of cells bearing del17p as well as increased sensitivity to the drug meayamycin which is an inhibitor of pre-mRNA splicing [65]. Mutation of *RPA1* is reported to contribute to the development of lymphoid tumours in mice [66], while reduced expression of this gene is linked to enhanced cell sensitivity to PARP inhibitors such as olaparib [67]. Although not represented within our panel of downregulated genes, LOH and haploinsufficiency of *MYBBP1A* in pancreatic cancer cells is reported to increase the activity of PARP inhibitors through a mechanism involving the eviction of a minimal pool of chromatin-bound MYBB1A [68].

**Table 1:** Common genes affected by LOH and haploinsufficiency in cells with del17p.

Gene	Essential CRISPR	Essential RNAi	TSG	Position on 17p	Chromosome band
*GLOD4*				757097	17p13.3
*TIMM22*	Yes	No		997129	17p13.3
*YWHAE*				1344275	17p13.3
*INPP5K*				1494577	17p13.3
*PITPNA*				1517718	17p13.3
*PRPF8*	Yes	Yes		1650629	17p13.3
*WDR81*				1716523	17p13.3
*SMYD4*			Yes	1779485	17p13.3
*RPA1*	Yes	Yes	Yes	1829702	17p13.3
*DPH1*			Yes	2030112	17p13.3
*SRR*				2303378	17p13.3
*SGSM2*				2337498	17p13.3
*METTL16*	Yes	No		2405562	17p13.3
*PAFAH1B1*	Yes	Yes	Yes	2593183	17p13.3
*CTNS*				3636459	17p13.2
*ITGAE*				3714628	17p13.2
*P2RX1*				3896592	17p13.2
*ATP2A3*				3923870	17p13.2
*ZZEF1*				4004445	17p13.2
*CYB5D2*				4143168	17p13.2
*UBE2G1*				4269259	17p13.2
*MED11*	Yes	Yes		4731428	17p13.2
*PSMB6*	Yes	Yes		4796144	17p13.2
*SLC25A11*				4937130	17p13.2
*SPAG7*				4959226	17p13.2
*NUP88*	Yes	Yes		5359668	17p13.2
*RPAIN*	Yes	No		5419641	17p13.2
*C1QBP*				5432777	17p13.2
*DERL2*				5471254	17p13.2
*MIS12*	Yes	No		5486285	17p13.2
*KIAA0753*				6578147	17p13.1
*ACADVL*				7217125	17p13.1
*DVL2*				7225342	17p13.1
*PHF23*			Yes	7235029	17p13.1
*GABARAP*			Yes	7240008	17p13.1
*CTDNEP1*	Yes	No		7243591	17p13.1
*ELP5*	Yes	No		7251416	17p13.1
*NEURL4*				7315628	17p13.1
*ACAP1*				7336529	17p13.1
*TMEM256*				7402975	17p13.1
*ZBTB4*			Yes	7459366	17p13.1
*POLR2A*	Not done	Yes		7484366	17p13.1
*SENP3*				7561919	17p13.1
*MPDU1*				7583529	17p13.1
*FXR2*				7591230	17p13.1
*SAT2*				7626234	17p13.1
*TP53*			Yes	7661779	17p13.1
*WRAP53*				7686071	17p13.1
*KDM6B*				7834217	17p13.1
*NAA38*	Yes	No		7856685	17p13.1
*KCNAB3*				7921859	17p13.1
*TRAPPC1*	Yes	No		7930345	17p13.1
*CNTROB*				7932081	17p13.1
*ALOXE3*				8095900	17p13.1
*VAMP2*				8159147	17p13.1
*PFAS*				8247608	17p13.1
*SLC25A35*				8287763	17p13.1
*KRBA2*				8356902	17p13.1
*RPL26*	Yes	No		8377516	17p13.1
*STX8*				9250471	17p13.1
*SCO1*				10672474	17p13.1
*ZNF18*				11977439	17p12
*MAP2K4*			Yes	12020829	17p12
*ELAC2*	Yes	No		12894929	17p12
*COX10*				14069490	17p12
*ZSWIM7*				15976560	17p12
*TTC19*				15999784	17p12
*NCOR1*				16029065	17p11.2
*COPS3*	Yes	Yes		17246616	17p11.2
*PEMT*				17505563	17p11.2
*TOM1L2*			Yes	17843511	17p11.2
*ATPAF2*				17977409	17p11.2
*GID4*				18039408	17p11.2
*DRG2*				18087892	17p11.2
*ALKBH5*				18183078	17p11.2
*AKAP10*				19904302	17p11.2

List of 76 common genes identified from the UpSet analysis presented in Fig. 4, reporting also on whether the gene was identified as “common essential” using either CRISPR or RNAi approaches (using the DepMap database) or as a Tumour Suppressor Gene (TSG). The position and cytoband of each gene on 17p is also provided.

As indicated in the paragraph above, LOH and haploinsufficiency will likely impact Tumour Suppressor Genes (TSGs), potentially helping to explain why cells with del17p can be easily selected and are more resistant to therapy. Of the 11 TSGs identified from the UpSet analysis (Fig. 4, Table 1), several are involved in epigenetic and transcriptional control of gene expression: *PHF23* functions as a H3K4me3 reader and deficiency in its expression, such as that associated with del17p, limits the ability of cells to differentiate and promotes their outgrowth as immature malignant cells [69]. *SMYD4* is another TSG involved in epigenetic regulatory control, heterozygous loss and downregulation of its expression is reported to facilitate breast cancer development as well as contribute to poor outcomes in patients with other tumour types [70, 71]. *ZBTB4* functions as a transcriptional repressor that is reported to regulate expression of the CDK inhibitor P21CIP1 during cellular stress, providing cells with an essential survival signal with which to overcome common front-line therapeutics such as vinscristine [72]. Other TSGs are involved in vesicular trafficking in cells; reduced expression of GABARAP in multiple myeloma cells bearing del17p is associated with inability to express eat-me signal calreticulin protein interactors leading to increased disease resistance to agents which induce immunogenic cell death in this disease [73], and reduced expression of *INPP5K* and *MYO1C* have been shown to contribute to tumour development in a rat model of endometrial carcinoma [74]. Finally, although not present within the list of genes affected by LOH and haploinsufficiency (Table 1), one study found that LOH and haploinsufficiency of the TSG *ALOX15B* contributed to the development of DLBCL in the Eμ-Myc mouse model of this disease [2].

**Fig. 5:**
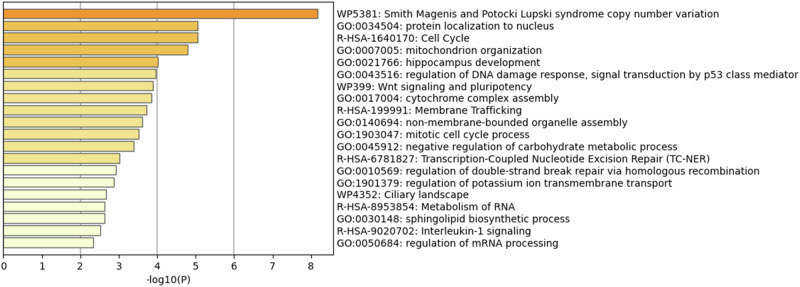
Enrichment pathway analysis of genes on 17p affected by LOH and potential haploinsufficiency. The panel of 76 genes identified from the UpSet plot analysis was subjected to enrichment pathway analysis using the Metascape tool (https://metascape.org). Reactome and GO terms are listed according to their probability score.

To more fully understand the impact of del17p on cells we performed an enrichment analysis of the affected genes listed in Table 1 using Metascape (https://metascape.org) [75]. Figure 5 shows that the enrichment term “Smith Magenis and Potocki Lupski syndrome copy number variation” is the most significantly represented. Such association can be expected given the majority of genes listed in Table 1 are located within the 17p13.1, 17p12 and 17p11.2 cytobands. However, this result is also surprising because gene density within these cytobands is less than what is observed in 17p13.3 and 17p13.2 [25]. One conclusion from this finding is that genes located in cytobands 17p13.1, 17p12 and 17p11.2 are functionally more likely to be affected by haploinsufficiency in cancers where del17p is a feature and therefore play a significant role in cell behaviour and disease outcome. Of particular interest is the enrichment of genes involved in mitochondrion organisation and in negative regulation of carbohydrate metabolic process. Mitochondrial biogenesis is reported to be increased in CLL cells with del17p [76], potentially in response to increased expression of p21 and Myc [77], and is consistent with the increased cell growth that is required to sustain the shorter lymphocyte doubling times associated with del17p CLL [78]. The rapid cell proliferation associated with del17p can also potentially be explained by haploinsufficiency of *MNT* which antagonises Myc [79]; downregulation of MNT protein is reported to facilitate MYC-driven transformation in Burkitt’s lymphoma [80] and is associated with adverse prognosis in renal clear cell carcinoma [81]. Further support for del17p resulting in increased cell proliferation is provided by enrichment of genes represented within the cell cycle. For example, reported reduction of PAFAH1B1 and resulting increased malignant cell growth in hepatocellular carcinoma [82] is likely a result of del17p that is commonly observed in this disease [83]. Similarly, reduced expression of the phosphatase CTDNEP1 is reported to facilitate aggressive tumour behaviour in medulloblastoma through amplification of Myc [84]. Finally, increased cell proliferation would be supported by changes to metabolic processes; del17p in CLL cells is associated with increased basal metabolism [85] resulting in increased lactate production and respiration, metabolic alterations observed to closely resemble the switch to aerobic glycolysis that promotes proliferation and survival of CLL cells exposed to B cell receptor (BCR) crosslinking.

## Cellular and biological consequences of 17p deletion – non-protein coding genes

**Fig. 6:**
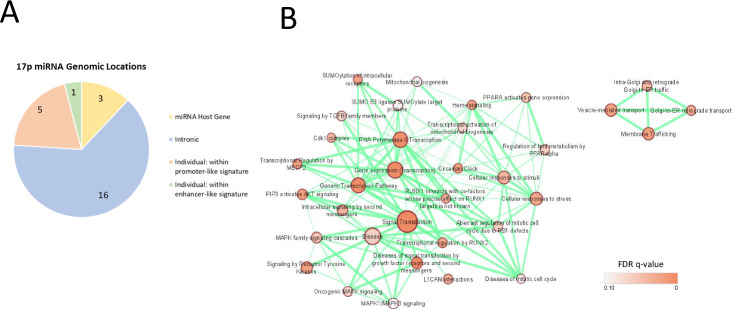
Positioning and REACTOME analysis of microRNA coding sequences located on 17p. **A** Pie chart showing the frequency of microRNA genes located within their own host gene, or within promoter or enhancer regions / intronic gene sequences of other genes. **B** Cytoscape EnrichmentMap visualisation of DAVID REACTOME pathway enrichment analysis of predicted targets with a target score of >90 for all 17p-derived miRNAs. The sizes of each node represent the number of genes within that particular pathway, while the node colour indicates the False Discovery Rate q significance value. The thickness of each connection indicates the number of genes shared between adjacent nodes (*p* < 0.01, *q* < 0.1, overlap threshold = 0.5).

Chromosome 17p also contains sequences encoding 25 miRNA, 292 long non-coding RNA (lncRNA), and 16 snoRNAs. In keeping with the high gene density observed in this region [25], the majority of these sequences are clustered within 17p13.3 – 17p13.1. 16 of the 25 miRNA encoding sequences are situated within introns of protein-coding genes, six miRNAs fall within enhancer or promoter-like sequences of genes, and three (miR-22, -497, and -195) are coded for within host long non-coding RNAs (Fig. 6A) and likely signify co-regulation and overlapping functions. The predicted targets of the 25 miRNA-encoding sequences on 17p were explored using miRDB (https://mirdb.org/). Targets with a prediction score >90 were visualised using Cytoscape [86] and EnrichmentMap [87], revealing significant enrichment of REACTOME ‘signal transduction’ as well as gene transcription pathways (Fig. 6B).

Importantly, miR-22, miR-195, miR-497, and miR-132 are recognised by the Cancer Dependency Map (DepMap, https://depmap.org/portal/) as tumour suppressors. miR-132, together with the other member of the same cluster (miR-212), has been implicated in B cell development and signalling. Specifically, loss of this cluster in mice exhibited reduced B cell apoptosis leading to increased numbers of total B cells in peripheral blood, bone marrow, and spleen [88], whereas enforced expression of miR-132 within the Eμ-Myc mouse-derived haematopoietic stem and progenitor cells limited the development of B-cell cancers [88], a finding that is consistent with other reports demonstrating tumour-suppressive properties of this miRNA [89, 90]. An important target of miR-212 and miR-132 is *FOXO3* [91]. Overexpression of this transcription factor is associated with short survival in cancers such as PDAC [92, 93] where del17p is present in a high proportion of cases [68]. *FOXO3* is also implicated in the pathogenesis of AML. Specifically, reduction in FOXO3 expression induces differentiation and apoptosis in AML cell lines and limits the development of AML in murine models [94]. Curiously, the gene coding for Activator of RhoGEF and GTPase, *ABR*, is located very close to the miR-132/212 cluster on 17p13.3 and is relevant because low expression of this gene in cells with del17p is reported to correlate with repressed malignant-cell differentiation and poor outcome in AML [95]. miR-22 is a recognised tumour suppressor in malignancies of the liver, lung and myeloid lineage [96–98], affecting processes such as epithelial-mesenchymal transition, cell differentiation and cell cycle (reviewed in [99]). The miR-497/195 cluster was first found reported to have tumour suppressive properties as early as 2011, when Li et al. found that the pair were significantly downregulated in breast cancer samples [100]. More recent studies have found that miR-497/195 is also under-expressed in other cancers [101, 102], low levels being associated with short patient survival. The prevalence of miR-497/195 under-expression is in keeping with the close proximity of the host gene to *TP53*, meaning that deletion of *TP53* would almost always be accompanied by co-deletion of the miR-497/195 cluster. Interestingly, the molecular targets of miR-497/195 (reviewed in [103, 104]) are different to those of miR-22, but the affected cellular processes, regulation of cell differentiation and of cell cycle, cell migration and tumour metastasis, are broadly similar. This suggests potential for a combined effect on these processes resulting from low expression of these miRs, as what might be expected if their host genes are affected by haploinsufficiency in cells with del17p.

Less is known about the lncRNA genes located on 17p. At least one, *PRAL* (p53 regulation-associated lncRNA), is reported to function as a tumour suppressor by regulating the ability of HSP90 to stabilise p53 protein levels [105–107]. Low expression of *PRAL* is reportedly associated with poor prognosis in non-small cell lung cancer [108], a finding similar to that in a more recent study showing that low *PRAL* expression in the malignant cells of multiple myeloma, especially those with 17p deletion, correlated with significantly shorter disease-free and overall survival [105]. This same study further showed that *PRAL* potentiates the anti-multiple myeloma effects of bortezomib by sponging miR-210 and inhibiting its ability to repress expression of bone morphogenetic protein 2 (BMP2). These findings suggest that low levels of *PRAL* confer bortezomib resistance in multiple myeloma cells bearing a 17p deletion. Consistent with its ability to regulate p53 protein levels, low *PRAL* expression resulting from del17p has also been reported to confer resistance to DNA-damaging agents in cell lines derived from hepatocellular carcinoma and lung cancer [106]. A second lncRNA linked to p53 stabilisation is *SNHG29* (small nucleolar RNA host gene 29) [109]. The role of this lncRNA as a tumour suppressor was recently described by Liu et al. [110] who demonstrated its influence on the genomic binding profile of the transcriptional activator EP300 and correlated low expression with aggressive tumour behaviour in AML. Other potential tumour suppressive effects include stabilising the expression of YAP and facilitating downregulation of programmed death ligand 1 (PD-L1) on tumour cells [111], and in maintaining cell stemness in AML by regulating expression of ETV6 target genes [112]. Importantly, *SNHG29* is a host gene for the small nucleolar RNA (snoRNA) genes SNORA49A, SNORA49B and SNORD65, all of which have been linked to the pathogenesis of ALL and AML [113–115], likely through their described contribution to tumorigenesis and cellular function by catalysing post-transcriptional modifications of ribosomal, transfer and mRNAs to influence protein production [116].

## Role of del17p in tumorigenesis

**Fig. 7:**
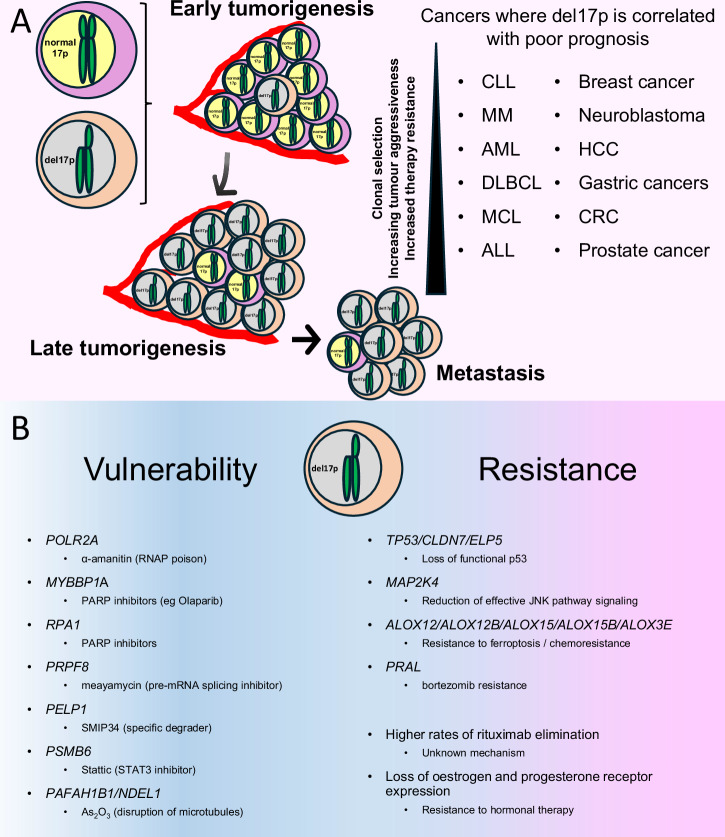
Role of del17p in tumorigenesis and therapy resistance/sensitivity. **A** Summary of the role del17p plays in tumorigenesis. del17p is an early event where selection processes dictate expansion of the clone which, in turn, adds to therapy resistance and increased aggressive behaviour / metastatic potential. The inset list indicates the cancer types where del17p is associated with aggressive tumour behaviour and poor clinical outcome. CLL chronic lymphocytic leukaemia, MM multiple myeloma, AML acute myeloid leukaemia, DLBCL diffuse large B cell lymphoma, MCL mantle cell lymphoma, ALL acute lymphoblastic leukaemia, HCC hepatocellular carcinoma, CRC colorectal carcinoma. **B** Summary of the impact of del17p on malignant cell vulnerability and resistance to therapy. Vulnerability / Therapy resistance likely results from LOH and haploinsufficiency of the listed cell-essential genes or TSGs, respectively.

Does del17p have a role in tumour development? The potential for such a role is great considering the density of TSGs on this chromosome arm [62]. Moreover, 8 proto-oncogenes are also present on 17p [117], but there are no reports of driver mutations within these genes that contribute to tumorigenesis in any cancer. On balance, however, del17p is unlikely to be a driver of tumorigenesis but more of a facilitator of other drivers, either by helping to make these drivers happen or by releasing restriction of their effects. Within this model, deletion of 17p is a discrete process within a single clone that occurs subsequent to its malignant transformation and relatively early in the evolution of the tumour. Evidence for this hypothesis is provided by studies showing the presence of del17p clones in monoclonal B lymphocytosis [118, 119], the precursor condition leading to CLL, in monoclonal gammopathy of undetermined significance (MGUS) and smoldering multiple myeloma (SMM) [120, 121], and of genomic instability in colorectal cancer which place del17p as an early event preceding CIN [46, 122]. Here, as discussed in this review, consideration must also be given to cell-essential gene haploinsufficiency which potentially weakens the ability of cells bearing del17p to effectively compete with other tumour clones. This means that expansion of such cells is accompanied by some sort of selective pressure resulting either from cell intrinsic factors, from therapeutic intervention (expansion of del17p clones is often observed at disease relapse in CLL [123] and MM [124]) and/or from the tumour microenvironment. Cell-intrinsic pressures may be caused by presence of driver mutations such as KRas(G12D) described for colorectal adenoma to carcinoma progression [122], whereas with tumour microenvironment CLL clones that are less able to take advantage of its ability to provide engagement of BCR, which is a key driver of CLL clonal expansion [11], are more likely to manifest as indolent disease [37]. Thus, where cell-intrinsic factors and/or tumour microenvironment is favourable, del17p clones will be selected and make up a greater proportion of the malignant clone in *de-novo* disease, ultimately contributing to shortened survival and increased disease aggressiveness as is observed associated with CLL and MM [16, 64]. This may be particularly relevant with respect to metastasis; dysregulation of miR-324-5p is reported to enhance the migratory and invasive abilities of MM cells by regulating the function of the SCF^β-TrCP^ E3 ligase [125], downregulated expression of GAS7 is reported to promote metastasis in neuroblastoma [126], and increased representation of malignant cells with del17p is associated with lymphnode metastases in intrahepatic cholangiocarcinoma [127] and with metastases in other cancers [128]. Figure 7A presents a proposed model of the factors contributing to the expansion of del17p clones, including the influence of therapy resistance as is discussed in the next section.

## Can del17p confer therapy resistance independently of p53?

In addition to the role of del17p in cancer biology, it is also important to consider how this aneuploidy may influence therapy response. Therapy resistance has been best studied in haemic malignancies, particularly in CLL, where studies employing serial sampling of patients show expansion of clonal populations of malignant cells carrying loss of 17p at disease relapse [20, 129]. These findings have since been expanded to include AML [130], MM [131, 132] and MCL [14]. Importantly, the size of the clone carrying del17p is important for outcome in MM [133] and in CLL [16], where patients who have more cells with this aneuploidy have increased resistance to therapy and a worse clinical outcome (Fig. 7A). *TP53* inactivation with loss of cellular processes mediated by wild-type p53 such as apoptosis, cell-cycle arrest and DNA repair, clearly plays an important role in mediating therapy resistance associated with del17p, and this has been reviewed elsewhere [134]. Additionally, as discussed earlier in this review del17p is associated with chromosomal instability in cancers and it is well known that chromosomal instability is a feature of therapy-resistant tumours as has been recently reviewed by Lukow and Sheltzer [135].

This section, therefore, only considers evidence linking del17p with other mechanisms of therapy resistance and is summarised in Fig. 7B. Interestingly, del17p is linked to higher rates of rituximab elimination in CLL, although the underlying mechanism is unclear [136]. In breast cancer, LOH at 17p13.3 was found to correlate with loss of oestrogen and progesterone receptor expression [137], with implications for hormonal therapy. Loss of MKK4, encoded by the tumour suppressor gene *MAP2K4* at 17p12, may also contribute to therapy resistance. In addition to potentially driving the selection of malignant clones bearing del17p due to the unusually linear gene-dosage phenotype of *MAP2K4* [138], inactivating mutations in these genes have been shown to impair JNK signalling and co-operate with mutant K-RAS to accelerate pancreatic ductal adenocarcinoma [139]. Through its ability to regulate JNK activity, MKK4 promotes Mcl-1 degradation [140], and loss of this effect might therefore explain why del17p is associated with shorter progression-free survival in CLL patients treated with the Bcl-2 inhibitor venetoclax [141]. In colorectal cancer, resistance to chemotherapy and adverse clinical outcome have been linked to low expression of *CLDN7*, a gene located at 17p13.1 that functions as a TSG in cells where p53 is functionally intact [142]. Finally, reduced expression of *ELP5*, which is also located at 17p13.1, is reported to confer gemcitabine resistance in gallbladder cancer [143]. *ELP5* regulates p53 IRES-dependent translation as part of the Elongator complex – a hexameric RecA-like ATPase that provides tRNA-specific binding sites.

Some TSGs located on chromosome 17p work in tandem with p53, and haploinsufficiency of these genes could therefore potentially allow cancer cells greater latitude to evade the effects of DNA-damaging chemotherapy. The high levels of cellular stress induced by chemotherapy activate p53, initiating downstream functions including cell death to maintain genomic integrity. One mechanism of p53-mediated cell death involves the downregulation of the cystine transporter *SLC7A11*, resulting in reduced cystine import and an iron-dependent form of cell death known as ferroptosis [144]. Key to the latter process is the formation of lipid peroxides. Notably, five of the six genes responsible for lipid peroxide production (*ALOX12*, *ALOX12B*, *ALOX15*, *ALOX15B* and *ALOX3E*) are located on chromosome 17p, four of them (*ALOX12*, *ALOX12B*, *ALOX15B* and *ALOX3E*) within 1Mbp of *TP53*. Reduced expression of any of the five ALOX genes on 17p has been shown to increase resistance to ferroptosis in various cell models [145–149], and selective downregulation of *ALOX15* has been linked with chemo-resistance in gastric and other cancers [150, 151]. Furthermore, deletions of *ALOX12* and *ALOX15B* have been shown to cooperate with p53 to facilitate tumorigenesis [2, 148, 152]. The observation that phenethyl isothiocyanate, a natural compound that lowers intracellular glutathione, induced the accumulation of reactive oxygen intermediates and preferentially killed CLL cells bearing del17p [153] suggests that it may be possible to activate the ferroptosis death pathway selectively in cancer cells with del17p.

Therapy resistance associated with del17p may also result from the impact of the deletion on tumour-microenvironmental interactions. Notably, loss of 17p in triple negative breast cancer has recently been reported to correlate with reduced tumour infiltration with cytotoxic T-cells and adverse clinical outcome [4]. A similar observation has been made in hepatocellular carcinoma, where del17p was associated with resistance to immune-checkpoint inhibitors [154].

### Can LOH of genes on chromosome 17p be exploited therapeutically?

Loss of 17p could create vulnerabilities in cancer cells that are potentially exploitable. Such synthetic lethality could be particularly effective given the survival/growth advantage associated with del17p subclones and their enrichment following chemotherapy. Notably, thirteen genes deemed essential for cell replication/survival located in 17p (Fig. 2B) [31, 32] show lower expression when there is LOH and are potentially targetable with drugs. This review has already discussed how *POLR2A* LOH leads to lower expression of RNA polymerase II (RNAP2) in del17p prostate and breast cancer cells, and how this renders such cells more sensitive to the cytotoxic effects of the fungal toxin α-amanatin [4, 9]. This vulnerability could be therapeutically exploited through antibody-drug conjugates, which are ideal delivery conduits for compounds such as α-amanitin which are highly toxic. This approach is now being tested in recent reports on the development of an antibody-drug conjugate that combines an anti-B cell maturation antigen (BCMA) antibody with α-amanitin (HDP-101) for the treatment of MM with del17p [155, 156]. HDP-101 is currently undergoing phase I clinical evaluation where early reports indicate that it is well tolerated in the dosage cohorts tested and may have clinical benefit [157]. Another example of synthetic lethality associated with del17p is the enhanced cell sensitivity to PARP inhibitors associated with LOH and haploinsufficiency of *PRPF8*, *RPA1* and *MYBBP1A* [67, 68]. Although not investigated in the specific context of 17p deletion, a recent report has described the targeting by proteasomal degradation of proline-, glutamic acid- and leucine-rich protein 1 (*PELP1*), an oestrogen receptor co-activator encoded at 17p13.2, in the treatment of ER+ breast cancer [158]. According to DepMap and cBioPortal, *PELP1* LOH is associated with haploinsufficiency in many tumours, raising the possibility that del17p might confer increased sensitivity to the PELP1 degrader, SMIP34. PELP1 also functions as a glucocorticoid receptor co-repressor, potentially explaining the unexpected effectiveness of therapy containing high-dose methylprednisolone in del17p CLL [159, 160]. LOH of the proteasome 20S Subunit Beta 6 (*PSMB6*), located at 17p13.2, also results in haploinsufficiency, and in myeloma the STAT3 inhibitor, stattic, is reported to inhibit PSMB6 protein function to restore sensitivity to the proteasome inhibitor, bortezomib [161]. Finally, there is evidence to suggest that the metastatic behaviour of tumour cells might be influenced by arsenic trioxide which displaces zinc from CLIP170 resulting in disruption of the PAFAH1B1/NDEL1/dynein microtubule complex [162]. Cancer cells with del17p may be more sensitive to this compound due to low expression of PAFAH1B1 and NDEL1 resulting from LOH/haploinsufficiency of the corresponding genes at 17p13.3 and 17p13.1, respectively.

### Models of 17p deletion

Despite the widespread occurrence of 17p deletion across multiple cancer types, study of its impact on tumour cell behaviour is restricted by the limited availability of isogenic cell lines that can be used for direct comparison. The first description of 17p13 deletion modelling was performed using a MICER (Mutagenic Insertion and Chromosome Engineering Resource) strategy to introduce *loxP* sites on to mouse chromosome 11B3 corresponding to a 4 Mb region that is syntenic to human 17p13.1 [2]. The heterozygous deletion produced through this approach was not lethal to embryonic stem cells but blocked embryonic development likely because of the essential role played by genes within this region during this process. Nevertheless, selective targeting of the 11B3 deletion into the mouse hematopoietic compartment accelerated cancer development in mouse models of B cell lymphoma and AML. These models were further used to elucidate *ALOX12* as a gene responsible for facilitating ferroptosis during p53-mediated tumour suppression [148] as well as characterise a role for the H3K4me3 reader *PHF23* as a tumour suppressor gene [69]. Hitherto, only two isogenic del17p cell lines have been reported, both of which were generated through Cas9 and sgRNAs targeting of *WDR81* (telomere) and *MAP2K3* (centromere) to remove the majority of 17p [4, 9]. These cell lines, derived from prostate (DU145) and breast (HS578T) cancer, respectively, have been used in xenograft models to demonstrate the efficacy of targeting haploinsufficient expression of *POLR2A* with antibody-drug conjugates containing α-amanitin. Interestingly, the DU145 del17p isogenic cell line was used in a CRISPR-based genetic screen which identified an essential role for RING box protein-1 (*RBX1*) and General transcription factor IIH subunit 1 (*GTF2H1*) specifically in cells with del17p [9]. This same study showed that RBX1, an E3 ubiquitin ligase, functions to ubiquitinylate RNAP2 to activate its RNA transcription activity and that blocking its expression in cells bearing 17p deletion makes them highly sensitive to the effects of α-amanitin. Together these studies demonstrate the power of an isogenic cell line approach to uncover new therapeutic targets, and call for the creation of further cell lines, representing other cancers, that can be used to investigate the mechanisms responsible for the rapidly progressive, drug resistant phenotype associated with this aneuploidy, as well as to explore novel therapeutic approaches based on synthetic lethality. These studies will likely involve generation of cell lines able to model specific patterns of 17p deletions representative of those found in diseases such as prostate cancer (Fig. 1E). Here, several technologies have been developed to study aneuploidy in cells, with most involving CRISPR/Cas9 to introduce DNA double strand breaks close to the centromere in order to generate chromosome arm deletions (reviewed in Truong, Cané and Lens [163]). With respect to deletion of smaller regions along 17p, caution will be required due to the presence of low copy repeats within chromosome 17 that make re-insertion of removed parts of the chromosome into the unaffected allele via non-allelic homologous recombination a possibility [25].

### Conclusions and future directions

There is clearly much to learn from focused research on the wider biological impact of del17p in cancer, over and above p53 dysfunction alone now that the required investigative tools are becoming available. A chief focus so far has been to investigate how del17p contributes to cancer development and whether this can be therapeutically exploited due to synthetic lethality created by haploinsufficiency of genes critical for tumour cell survival. An important caveat here is that the contribution of del17p to disease pathogenesis has only been modelled in DLBCL and AML, and that essential genes are likely to vary with tumour type [31, 32]. Consequently, there is need to develop additional models representing other cancers where this aneuploidy is prevalent, such as pancreatic ductal adenocarcinoma and colorectal carcinoma. This might extend to further interrogation of existing or the development of new mouse models to study the impact of del17p on how this aneuploidy affects the pathogenesis of cancer. Such models have the potential to yield significant insight because of the high degree of synteny between mouse chromosome 11B and human chromosome 17, particularly with respect to 17p (Fig. 3) [25]. As already discussed within this review, seminal work by the Lowe group has used this model to show how del17p accelerates disease progression in DLBCL and AML [2, 69]. Importantly, the deletion created by this model, targeting mouse 11B3 (human 17p13.1) encompasses 31 of the 76 genes that are downregulated in cells with del17p (Table 1), this suggests that further interrogation of this model will yield interesting insight. Study of del17p within a mouse model now needs to be extended to other cancers, an approach that will need to use tissue-specific activation of Cre as has been previously used to create cardiac tissue-specific deletions of mouse chromosome 11 [60] because of the lethality associated with deleting mouse embryonic stem bearing loss of particular regions of chromosome 11. Additional del17p isogenic cell lines need also to be created for comparative studies of how different cancers adapt to selective pressures arising from the tumour microenvironment and exposure to different therapies. The data on deletion size for the cancers presented in Fig. 1 indicates that deletion of the entire short arm is common but not universal, and that tumour-specific models might be required. An interesting hypothesis that could be explored in mouse and isogenic models is that malignant cells harbouring del17p shape their microenvironment in a specific way, a notion made all the more intriguing by a recent review of how driver mutations impact the tumour microenvironment in lung cancer [164]. Finally, if del17p can be detected at single-cell resolution (e.g. using gene signatures associated with del17p) to differentiate between 17p-intact and -deleted cells, it should be possible to investigate how these different cell populations interact with each other within tumours. In summary, del17p is notable for its occurrence across a range of tumour types, its association with aggressive disease behaviour, and its propensity for clonal selection by therapy. Although del17p is strongly associated with *TP53* mutation, the association is only partial and it is clear that its deleterious effects result from p53-dependent and/or independent mechanisms. Understanding these mechanisms promises to identify new drug targets, while therapeutic strategies based on synthetic lethality due to LOH/haploinsufficiency of essential genes on 17p set the scene for a Shakespearian fate whereby del17p cancer cells are hoist by their own petard.
